# Personalism or party platform? Gender quotas and women’s representation under different electoral system orientations

**DOI:** 10.1371/journal.pone.0257665

**Published:** 2021-09-23

**Authors:** Aliza Forman-Rabinovici, Lilach Nir

**Affiliations:** 1 Department of Sociology and Anthropology, Tel Aviv University, Tel Aviv, Israel; 2 Department of Communication and Department of Political Science, Hebrew University, Jerusalem, Israel; Bucharest University of Economic Studies, ROMANIA

## Abstract

Underrepresentation of women in politics is a matter of great concern to social scientists, citizens, and policymakers alike. Despite effort over the past decade to ameliorate it with gender quotas of different types, scientific research provides a mixed picture on the extent to which quotas can close these gender gaps under different conditions. We approach this puzzle by focusing on the orientation of electoral systems—*candidate*-centered vs. *platform*-centered—as a context that conditions the effect of quotas on representation. Our analyses of 76 countries’ electoral rules and legislatures show that contrary to expectations, it is in *candidate*-oriented systems that quotas facilitate stronger effect on women’s representation. Even after considering proportional representation, district magnitude, human development, or labor-force participation as alternative explanations, we show that quotas foster greater increases in gender representation in candidate-oriented systems. The broader implications are that in electoral systems that tend to have larger gender gaps, quotas have a substantial contribution to equal representation.

## Introduction

Though women make up half the population, on average around the world women hold only 25% of legislative seats. In other words, only one in four seats are held by a woman in national legislatures. To ameliorate this underrepresentation of women, electoral gender quotas have been proposed over the years. Quotas at the party- and the legislature level purposefully aim to advance female candidates. Where are quotas more effective in accomplishing this aim? Is it in electoral systems that produce greater platform-oriented commitment of voters? Or, in contrast, in systems that center on individual candidates’ personal qualities? Programmatically-oriented systems are electoral systems that place a high value on the candidate’s loyalty to the party platform, and voters focus more on the party platform than on an individual candidate’s idiosyncrasies. In candidate-oriented systems, on the other hand, the emphasis is on the individual candidate’s identity, and voters tend to focus on candidate personality to greater extent than party platform. In such systems, there is more importance given to seniority, uninterrupted careers, and experience [1].

As we explicate below, there are reasons to expect that either system orientation can condition the effect of quotas to better represent women. Empirical evidence, however, is either mixed or lacking on the factors that facilitate a stronger correlation between quotas and women’s increased representation in parliaments. Drawing on these bodies of important previous literature, we develop testable hypotheses on the conditional effect of quotas on representation in different systems. In the next section, we briefly review past studies on gender quotas efficacy. Following that section, we develop different explanations that lead to competing expectations on the conditional effects of programmatic vs. candidate-oriented system differences.

### Electoral gender quotas

Gender quotas are a significant factor in how women become representatives [2–4]. As of 2015, mandatory gender quotas exist at the federal level in 73 countries, constituting 37% of the world’s nations [5]. While first adopted in the 1970s by individual political parties in Western Europe, they are common today in Latin America, Africa, the Middle East, and Europe [6, 7]. Both developed and developing countries, in short, adopted quotas.

Quotas take three main forms: reserved seats, legislative quotas, and political party quotas. In a reserved seat system, a certain number of places in the legislature are designated for women, regulating the number of women elected. This type of reform is often constitutional or written into electoral law. Legal candidate or legislative quotas are the newest type of quota and require parties to nominate a certain percentage of women on their electoral slate. Voluntary party quotas, the most popular type, aim to increase the proportion of women on a party list. Unlike other quota policies, party quotas are adopted on a voluntary basis by individual parties. They can exist simultaneously with other types of quotas [8–10].

The research conducted on quotas to date has focused mainly on the adoption process and on factors contributing to quota efficacy. There are four main explanations for why quotas are adopted: women’s mobilization, political elites adopting quotas for strategic reasons, as a result of emerging local notions of equality, and international and transnational influences and policy diffusion. Within the category of party quotas, parties are more likely to adopt quotas in systems where there is a prototype quota policy at the system level, where there is a greater number of women in the upper echelons of the party, and where the party holds leftist positions [9, 11].

Comparative studies have shown that quotas do increase the number of female representatives [4, 6, 10]. However, quota policy success and efficacy vary as a result of policy design, institutional context, political will, political actors, and other influences. The enforcement mechanisms of the quota policy are especially crucial to success. The greater the costs of non-compliance, the more likely quotas are to be effective in increasing the number of women in the legislature [2, 7, 9, 12, 13].

Gender quotas are contingent on electoral system mechanisms, and differences in the electoral system can determine the efficacy of gender quotas. Party list type, district magnitude, and electoral system all influence quota efficacy. On the other hand, quota legislation has been found to increase the number of female representatives *regardless* of the type of party list [10, 14]. Quotas were found to be most effective in large districts with a closed-list form of proportional representation [15, 16]. Jones and Navia, for example, found that quotas did not have an effect on the percentage of women elected in open-list proportional systems, but they did have a positive effect in closed-list proportional systems [15]. The character of party bureaucracy has also been found to influence the efficacy of quotas as parties with a more bureaucratized selection process are better at implementing legally mandated quotas [17]. Although system characteristics have been found critical in determining quota efficacy, the effect of the candidate- versus programmatic orientation of the system remains to be theorized and tested.

### Theoretical considerations: Quotas under different electoral systems

Electoral system scholars distinguish between systems with a programmatic orientation and those with a candidate orientation. Programmatically-oriented systems are electoral systems that place a high value on a candidate’s loyalty to the party platform, and voters focus more on party platform than on the individual candidate’s persona. In candidate-oriented systems, in contrast, emphasis is placed on the individual candidate’s identity, and voters focus on candidate persona and positions more than on the party platform. In such systems, more importance is placed on seniority, uninterrupted careers, and experience [1].

**Fig 1:**
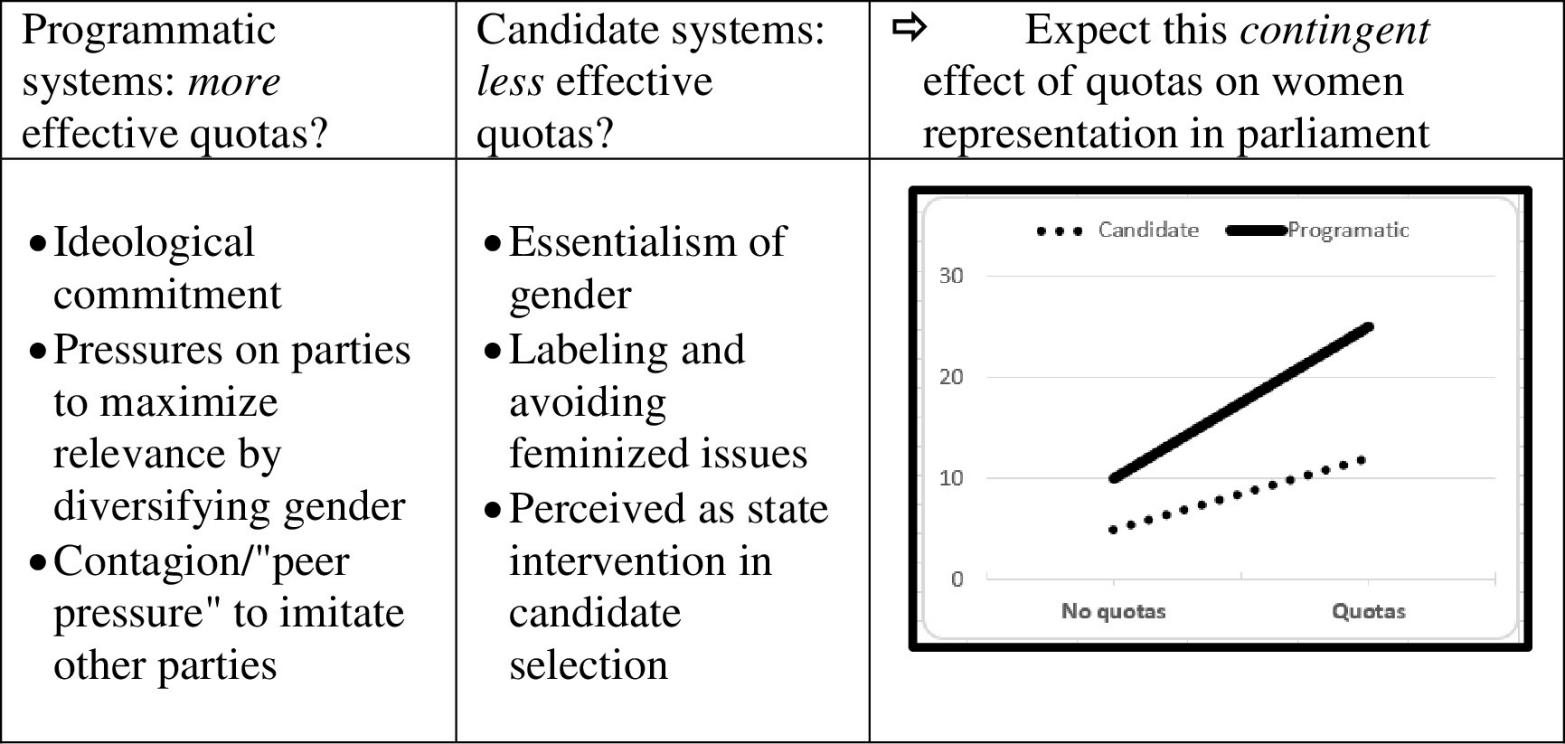
Rationale and hypothesized expectations: Stronger effect of quotas on representation in programmatically oriented systems.

A cursory inspection of the previous literature would suggest that the presence of quotas will correlate with a greater increase in female representation in *programmatically* oriented systems, compared to candidate-oriented ones. As we explicate below, certain features of programmatic systems facilitate quota efficacy, and certain criticisms of quotas in general are even more relevant in candidate-oriented systems. We discuss them and summarize them in Fig 1 below.

The first reason that quotas might correlate with a greater increase in representation in programmatically oriented systems is that these systems have certain characteristics that facilitate women’s political representation, [1, 18, 19] which also lower the cost of compliance with quota policy, and perhaps even raise the cost of noncompliance. Three, in particular: issue-based focus, pressures to diversify, and modeling other parties, are characteristics of programmatically oriented systems that lead us to expect a quota “advantage” in these systems.

The first feature of programmatic systems that could support quota efficacy is the emphasis these systems place on list and issue-based platform over candidate persona. In that context, women and minorities do not stand out as being nontraditional candidates as much in lists, making it less of a risk for parties to include them [20, 21]. This emphasis on lists also means that there is less emphasis on seniority and uninterrupted careers for any individual candidate [22]. For women, who often take time off for family obligations, such systems offer better chances of success, as well as create less risk for parties to adhere to quota policy, and put female candidates in realistic positions in their lists.

A second feature of programmatic-oriented systems that could support quota efficacy is that parties in programmatic systems have more pressure to diversify lists. Parties need to maximize their relevance, and the exclusion of any sector could signal discrimination and hurt general appeal [18, 22]. This encourages parties to make diverse lists that will attract a variety of demographics [18], thus automatically including women. Greater district magnitude, which is more prevalent in programmatically-oriented systems, also contributes to the ease and necessity of diversifying lists [18, 21]. This coordinates well with the aim of quotas and might mean that parties will be more open to facilitating goals set by quotas and sense higher potential reward for complying with quotas.

Finally, parties in programmatic systems are more likely to advance effective quotas as a result of peer pressure or a contagion effect from other parties in their system than are parties in candidate-oriented systems [21]. Parties in programmatically oriented systems are very sensitive to peer pressure, and as a result, compliance with mandatory quotas, or the adoption of quotas by a smaller fringe party is more likely to lead to similar behavior throughout the party system than would be the case in candidate-oriented systems. These expectations from past literature are summarized in Fig 1 below.

Common criticisms of quotas imply that they would be less effective in *candidate*-oriented systems. Quotas may contain “dangers of essentialism” [3]. This danger is based on the conviction that the individuals represented through quotas have some essential traits that define them and render them unable to be represented adequately by those without such traits. If only women can represent women, this would imply that only men can represent men. Quotas, as this line of argument goes, can therefore reinforce identity stereotypes, making it more difficult for women to be effective politicians.

Second, quotas could also create either a “mandate effect” or a “labeling effect.” A mandate effect would occur when female legislators elected through quotas feel an obligation to act on behalf of women. Quotas though could have a “labeling effect” in which women elected through quotas are perceived as less experienced and less autonomous. This would limit elected women’s ability to push through legislation and garner public support. It might also push these representatives to avoid women’s welfare issues in order to prove that they can behave like their male counterparts [8].

Third, within democratic contexts, quotas can also be perceived as blatant state intervention, a violation of individual freedom and democratic principles, and a violation of principles of equal opportunity [7]. This perceived weakening of democracy might hurt a candidate’s ability to get elected or to perform as an effective political representative.

These criticisms of quotas focus on the impact quotas could have on individual candidates and their efficacy. As candidate-oriented systems put greater spotlight on the individual, there is a greater chance of such candidate-focused weaknesses and criticisms existing in those systems than in programmatically oriented ones. These points imply that quotas might hurt both the legitimacy of female representatives as well as motivation of the parties to comply with quota policy. If parties sense a cost for promoting female representatives through quotas, they might be reluctant to advance the substantive aim of gender representation.

Given potential enhancing effects of programmatically oriented systems, and the criticisms that might be more relevant in candidate-oriented systems, we hypothesize that the correlation between gender quotas and increased women’s representation will be stronger in programmatically oriented systems (see Fig 1 above). In other words, we expect a significant interaction term between *system* characteristics (programmatic vs. candidate orientation) and gender quotas in their joint effect on gender representation. Formally put:

*Hypothesis*: Electoral gender quotas have a stronger impact on women’s share in parliament in programmatically (vs. candidate) oriented systems

## Method and measures

### Variables

#### Outcome variable

Our dependent variable is ***women’s representation*** in parliament, measured by the percentage of seats in the lower house of parliament that were occupied by female legislators. We use the Inter-Parliamentary Union’s 2015 Women in Parliaments Report, which contains data for over 180 countries [23].

#### Independent variables

Our main independent variables are ***system orientation*** and electoral gender ***quotas***. In order to measure candidate or programmatic orientation of an electoral system, we used the *Electoral Systems and the Personal Vote* database [24] *pers_rank* (personalism) index. This variable was created by Johnson and Wallack with Carey and Shugart’s ranking of personal vote incentives in mind; a score from 1 to 13 codes electoral systems for the extent of incentives to cultivate a personal vote and how much the electoral competition places emphasis on parties versus individual candidates [25]. It takes into consideration the nature of party ballots, how votes are cast votes and how votes are pooled.

The authors rank thirteen feasible combinations of three variables common to all electoral systems: degree of party control over the ballot, the degree to which votes are pooled, and the number and types of votes that citizens can cast, and each of these can take values of 0, 1, or 2; the larger the number, the greater the incentives to cultivate a personal vote. The final index of ***system orientation*** ranges from 1 to 13; the high anchor indicates systemic emphasis on individual candidate profile. The *pers_rank* dataset is time-series, and its most recent year is 2005.

Our second independent variable is electoral gender ***quotas***, dichotomized depending on whether the country had a quota in the last election held before 2015 (parliamentary data collection year) or not. Countries were coded as having an electoral quota if they had a mandatory legislative quota, or if at least 30% of parliamentary seats are held by parties with gender quotas. The 30% cutoff point is based on past work, which suggests 30% as the minimum threshold for minorities to exert influence [26, 27]. If 30% or more of the parliament seats were held by parties with a quota, these parties, their party culture, and their representatives were exerting some level of system-level influence. Reserved seat quotas were excluded from the analyses; electoral system should not matter in a reservation system. Therefore, the dichotomous variable represents electoral systems in which there is a quota that is either a mandatory legislative quota, or a voluntary quota in parties that hold at least 30% of the legislature. (39, 51% of the 76 countries had quotas). Voluntary and mandatory quotas were grouped together due to small group size.

#### Control variables

To account for different characteristics of electoral systems that are known both to impact quota efficacy and the rate of female representation, we include the following variables. ***Proportional representation*** (PR) electoral system was coded dichotomously; PR systems are considered both more hospitable to quotas and to women’s representation. Because there is an overlap between system-orientation and type of electoral system, i.e., programmatically-oriented systems tend to be PR systems, we estimate their contribution separately. ***District magnitude*** was another control; DM is known to impact women’s representation, with larger districts encouraging more diverse lists and female representation [10, 15, 16].

In addition to these institutional features, we controlled for known predictors of women’s share of seats in parliament. The ***Human Development Index*** (HDI) was included to control for differences between countries associated with economic and social development. The HDI is composed of measures of life expectancy, education, and per capita income indicators [28]. Development status and economic strength are often associated with gender equality; this allows us to partial out development and gender equality from institutional features’ effects on women’s representation [29–32].

An additional control we employed was for ***women’s workforce participation***, measured as a ratio of women to men’s workforce participation. Studies find a consistently positive association between women’s workforce participation and women’s political representation [33–37]. Including workforce therefore controls both for a known predictor of political representation, and a different indicator of general gender equality within a country. All control variables were added in stages, to observe how results changed with the addition of each variable. Variables for the size of the quota and enforcement mechanisms of quota policy were also included in models not featured in the paper. These variables were statistically insignificant, and did not significantly change results.

### Analytical strategy

To test whether the effect of quota on representation varies by systems, we examine their additive contribution as well as their interaction. We aim to include a maximum number of countries (N = 76). We limit to countries that are in the upper tier of the rankings of democracies or partial democracies (4 and higher on the polity scale). This excludes countries that had blatantly biased or fraudulent elections without overly limiting our sample. The polity index assigns scores ranging from −10 to 10, with −10 being a hereditary monarchy and 10 being a consolidated democracy [38]. Scores are composites that reflect characteristics of executive recruitment, constraints on executive authority, political competition, civil rights and civil liberties, and institutionalized qualities of the governing authority. Based on Marshall et al.’s own scale application, scores of 7–10 are considered democracies [38].

To ensure consistency with the index of personalism candidate orientation from 2005, we only included countries that had not undergone any major electoral changes between 2005 and 2015. Again, we used the polity index to exclude countries that had a major score change between 2005 and 2015 or that had moved to a different regime categorization in the specified years. While this does not perfectly reflect institutional changes, it stands as an indicator for major changes, which would also imply changes in an electoral system.

In the Results section, we report in Table 1 the estimations for all countries (N = 76), and in Table 2 the estimation for each type of system orientation, candidate- or programmatically oriented. Countries scoring below 7 on the *pers_rank* index were coded as programmatically oriented, and countries with scores of 7 or higher were coded as candidate-oriented. The two groups of models for candidate and programmatically oriented countries include four models. With each model, an additional or alternative control is added.

**Table 1:** Women’s representation in parliament (pooled).

Model	1	2	3	4	5	6
Quotas (0, 1)	-1.36 (4.78)	-1.51 (4.81)	10.27 (12.70)	-1.35 (5.39)	2.52 (3.41)	2.38 (3.43)
PR: Proportional Representation (0, 1)	-3.30 (3.96)	-3.11 (3.97)	3.54 (7.73)	-3.06 (4.06)	-2.79 (4.10)	-2.56 (4.14)
District Magnitude	--	-.026 (.042)	-.024 (.042)	-.024 (.048)	--	.024 (.042)
Personalism (candidate orientation) index	-1.75 (.693)**	-1.77(.697)**	-.897 (1.11)	-1.76 (.710)**	--	--
Quotas x Personalism index	1.16 (..656)*	1.16 (.659)*	..114 (1.23)	1.15 (.697)*	--	--
Candidate orientation (0,1) dichotomized	--	--	--	--	-11.74 (5.08)*	-11.77 (5.10)*
Quotas x Personalism dichotomy	--	--	--	--	7.16 (4.86)	7.19 (4.88)
Quotas x PR			-9.05 (9.03)	--		
Quotas x DM			-	-.007 (.010)		
R^2^	24%	25%	26%	25%	23%	24%
Adjusted R^2^	20%	19%	19%	18%	19%	18%

*Notes*: Entries are unstandardized regression coefficients; SEs in parentheses. N = 76 countries.

*p < .05

**p < 0.01

***p < 0.001 (one-tailed tests). HDI = Human Development Index

**Table 2:** Women’s representation in parliaments, by system orientation.

	Candidate				Programmatic			
Model	1	2	3	4	5	6	7	8
Quotas (0, 1)	8.14 (3.45)*	7.68 (3.50)*	7.16 (3.48)*	9.62 (3.65)**	6.37 (3.47)*	6.45 (3.57)*	6.54 (3.70)*	4.14 (3.97)
PR: Proportional Representation (0, 1)	-1.09 (4.44)	-.273 (4.55)	.194 (4.50)	2.98 (4.92)*	2.32 (9.48)	2.41 (9.62)	2.48 (9.80)	3.34 (9.56)
District Magnitude	--	-.053 (.059)	-.049 (.059)	-.022 (.061)	--	.008 (.056)	.010 (.058)	-.042 (0.68)
HDI	14.18 (9.04)	15.52 (9.19)*	20.48 (2.10)*	15.03 (9.00)	46.17 (15.78)**	46.49 (16.18)**	45.49 (18.27)**	49.69 (16.20)**
Women Work Participation	--	--	.200 (.145)	--	--	--	.037 (.298)	--
Quotas x DM				-.296 (.189)	--	--	--	.150 (.118)
R^2^	24%	26%	30%	31%	22%	22%	22%	26%
Adjusted R^2^	18%	17%	19%	21%	15%	12%	10%	14%

*Notes*: Entries are unstandardized regression coefficients; SEs in parentheses.

*p < .05

**p < 0.01; ***p < 0.001. HDI = Human Development Index; DM = District Magnitude

A full list of countries categorized based on orientation and quota status can be found in the S1 Table.

**Fig 2:**
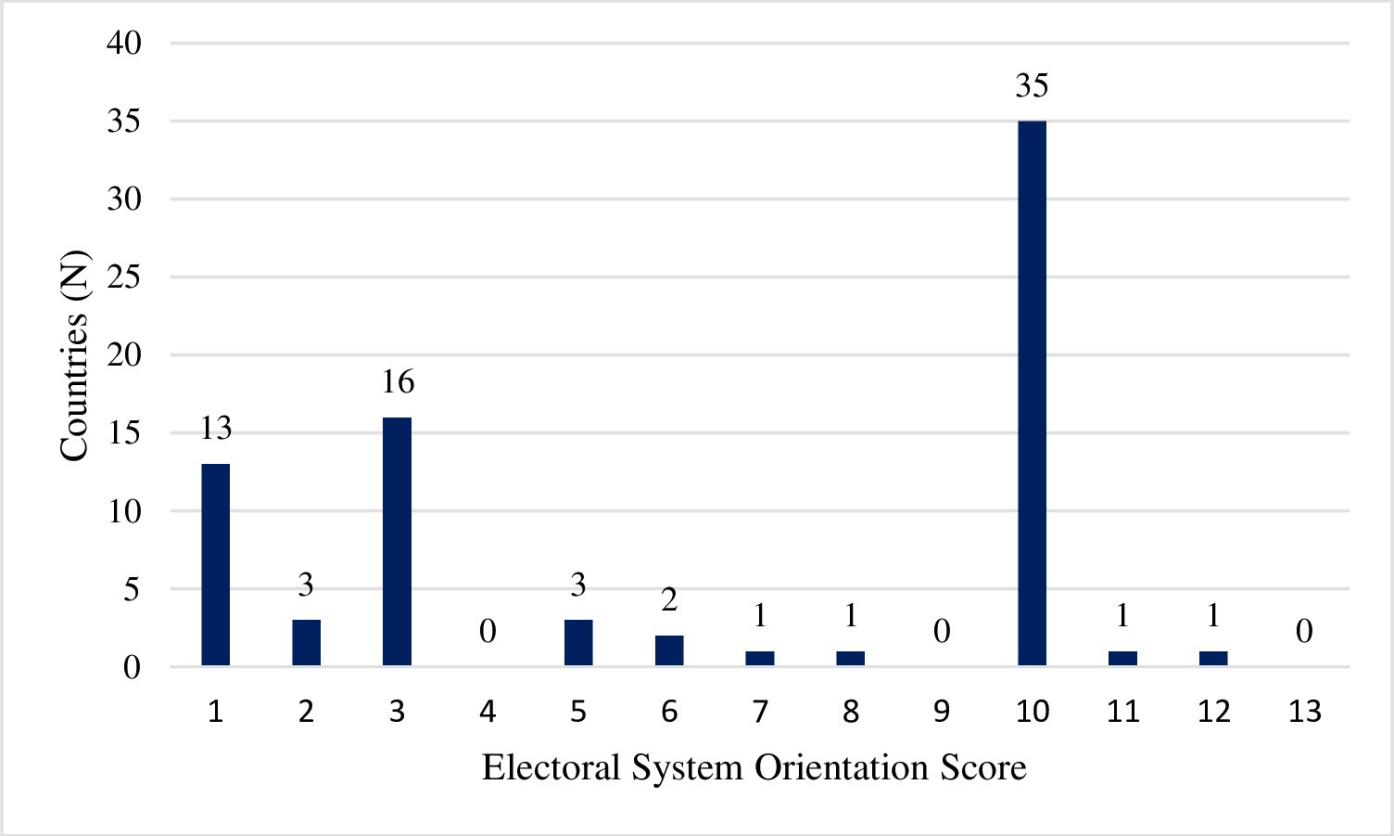
Electoral system orientation distribution.

In dividing countries into programmatically and candidate-oriented countries, we recognize that turning an ordinal scale into dichotomous categories could be problematic as it might place fairly similar countries into two separate categories. For example, countries with scores of 6 and 7, respectively, would be coded differently no matter how similar they are in reality. That said, this data lends itself fairly well to dichotomous categorization. Fig 2 shows the distribution of pers_rank scores. Of the countries in the study, only 9% fell in the middle range between 4 and 9. Most programmatically oriented countries fell between 1 and 3, and most candidate-oriented countries had a score of 10. The data itself had a bimodal distribution that lends itself well to our two categories. S2 Table (see supplement for reviewers) offers some descriptive statistics that demonstrate the small variance in the binary distribution. Programmatically oriented countries had an average score of 2.54, with a standard deviation of 1.5. Candidate-oriented countries had an average score of 9.9, with a standard deviation of 0.69.

## Results

Do gender quotas increase the chances of women’s representation? Are these better chances in programmatically-oriented systems or in candidate-oriented ones? Descriptive statistics suggests a system-quota contingent effect (see S2 Table). Indeed, countries without quotas had, on average, 15.7% women in parliament, while countries with quotas had an average of 24.7% women in parliament. In programmatic systems, where those without quotas had an average of 25.2% women in parliament. Countries with quotas had an average of 27.2% women in parliament. In candidate-oriented countries, there was an average of 9% more women in parliament in the presence of quotas. Put differently, having quotas (vs. absent quotas) affect the rate of representation more dramatically in candidate-oriented countries.

To formally test the study hypotheses, we ran multiple regression models. Table 1 below displays the results of all six pooled models, regressing women’s representation on several explanations. In all but one model, the system orientation was found to be significant. The average coefficient size for personalism throughout these four models was -1.76, implying that for every 1-point increase in the *pers_rank* scale, there was a 1.76% decrease in female parliamentarians. The results were replicated in models five and six which used the dichotomous variable for system-orientation. These two models had an average coefficient of -12.19 for the dichotomous variable, implying that countries coded as candidate-oriented are predicted to have 12.19% fewer women in parliament than programmatically oriented countries. In the *pers_rank* models, quotas did not achieve significance, but the interaction between quotas and system orientation was significant. As systems became more candidate oriented, quotas had a positive effect on the ratio of female parliamentarians (Model 4). The interaction of quotas with dichotomized versions of the continuous personalism index, on the other hand, were not significantly associated with representation (Models 5, 6).

**Fig 3:**
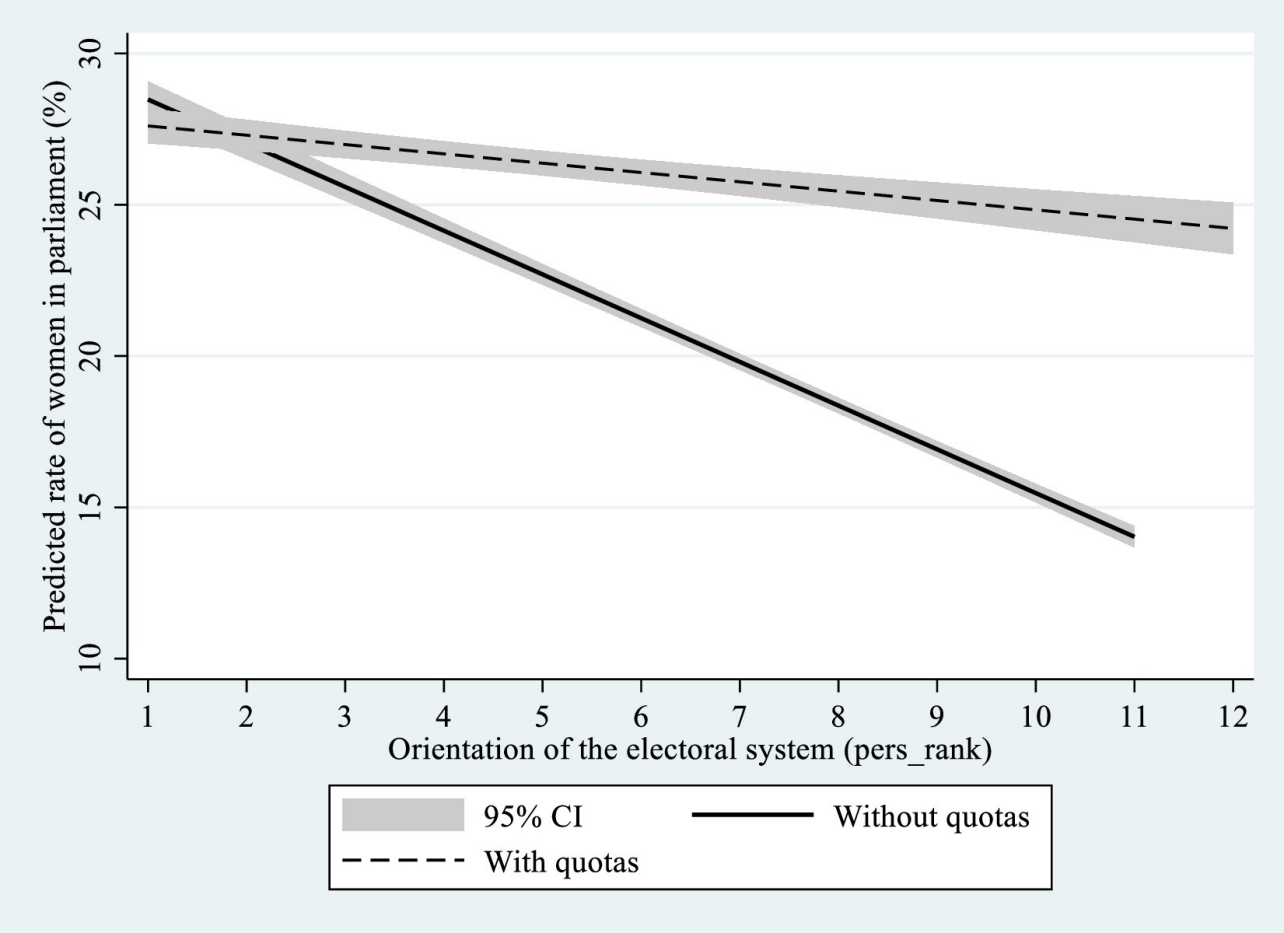
Women’s representation, by system orientation and quotas.

Fig 3 below graphs the contingent association between quotas and personalism (programmatic- vs. candidate-orientation index; see Table 1, Model 1). The chart plots predicted values of women’s share in parliament, by the presence and absence of quotas over the range of possible pers_rank scores. As seen in Fig 3, the contribution of quotas to representation is different over the range of scores, with the gap between quota and non-quota countries becoming more pronounced as the system becomes more candidate oriented. Countries with low pers_rank scores are expected to have relatively similar rates of women in parliament, whether or not quotas are in place. On the other hand, as the system gets increasingly candidate oriented, quotas have a significant expected impact on the rate of women. If a quota is in place, there will not be a big difference between a candidate-oriented system and programmatic systems with and without quotas. If there is no quota system in place, a very candidate-oriented system is predicted to have significantly less women in parliament.

To further probe this counter-intuitive, counter-hypothesized finding, we estimated the models separately. Table 2 displays the estimation for candidate-oriented and programmatically-oriented systems separately (*N*s = 39 and 37, respectively). As seen below, quotas were significantly correlated with an increased number of female representatives in all models but one. The regression coefficient for quotas was also slightly higher in candidate-oriented systems.

Note that other known predictors—proportional representation, district magnitude, women’s labor force participation—were *not* systematically associated with increased women’s representation in parliaments, once both system orientation and quotas were held constant in the estimation. Human development, as measured by the HDI, was the only other consistent predictor of women’s representation.

We used post-estimation tests to compare between the coefficients of the quota variables between models in different systems. There was no significant difference in quota coefficients in the first models, 1 and 5, χ^2^ (1, N = 76) = .13, p = .7291, ns; nor was there a difference between the second respective models, χ^2^ (1, N = 76) = .06, p = .8081; the third models, χ^2^ (1, N = 76) = .01, p = .9039; and the fourth respective models, χ^2^ (1, N = 76) = 1.05, p = .3057; t-tests of the differences between coefficients similarly revealed no significant differences in quota coefficients.

## Discussion

Do quotas have a differential effect on women’s representation, depending on systemic features? In this study, we tested an expectation derived from the scholarly literature, that *programmatic* (versus candidate-centered) systems will show a stronger correlation between quotas and representation. Empirical findings we presented show that, contrary to expectations, gender quotas have a larger impact on women’s representation in *candidate*-oriented systems. Both separate and pooled models showed the relative contribution of quotas, after different competing explanations, such as human development, district magnitude, or women’s labor force participation, were taken into account. That said, our findings do not provide an unequivocal answer

Why do results disconfirm the expectations we derived from past literature? One possible reason that in candidate-oriented systems, the competitive and candidate-focused characteristics of these systems may give quotas more latitude to influence representation, compared to systems that are already “female-friendly.” Women might be already at a distinct disadvantage in candidate-oriented systems, which emphasize uninterrupted careers and higher personal cost of campaigns. Policy to advance women will have a more significant impact compared to other systems because there will be more room to influence and create change than in a system that already accommodates female representation.

It could be argued that these differences in the systems could also be attributed to the fact that programmatically-oriented systems tend to be proportional representation (PR) systems. Therefore, the hospitable environment for women politicians might be explained by the type of system, rather than its orientation. If this were true, we would expect that system orientation would not correlate with representation, once proportionality was in the estimated model. Findings showed otherwise, suggesting that orientation explains quota efficacy above and beyond the impact of electoral system. In other words, our findings would imply that perhaps the effect that was attributed in previous studies to proportional representation systems overlapped with the quality of their being more programmatically oriented.

A second reason that candidate-oriented systems might be more sensitive to the effects of quotas is that women’s difficulty in gaining elected positions in candidate-oriented systems might largely be because of their difficulty in getting nominated for candidacy [20]. Some studies have found that having once appeared as a candidate, women have as good a chance as men at winning an election [36, 37]. Gaining the nomination is a greater challenge in candidate-oriented systems, where party elites are less inclined to advance nontraditional candidates. Gender quotas sidestep this challenge, promising women the seat or nomination.

Third, because of the emphasis placed on individual candidates in candidate-oriented systems, gender quotas and increased female representation may be significant in terms of symbolic representation and result in a greater impact, that of increased female representation. Female role models, in politics and in other fields, can inspire other women to follow a given path [39, 40]. Thanks to quotas, more women enter politics and their presence inspires more women to run for office. This will be more pronounced in *candidate*-oriented systems, where the individual candidate, man or woman, are more salient in the campaign.

Fourth, candidate-oriented systems are generally more competitive than programmatically-oriented systems. This is due to a variety of reasons, including smaller district magnitudes, more focus on long-term careers, and more expensive campaigning for the individual candidate [41]. By mitigating the characteristics of competitiveness for female candidates, while leaving the same competition for male candidates, quotas could have a more pronounced impact in systems with these higher levels of competition.

For advocates of quotas, this means that the orientation of the electoral system is a key factor in quota success. It also means that in candidate-oriented systems, where women tend to be underrepresented, quotas might be especially impactful. The body of literature contains discussion of a wide range of conditions under which quotas are most effective. We add to this literature the condition of candidate-oriented systems.

These findings are not unequivocal though. While in our models, on average quotas had a greater impact in candidate-oriented systems and the interaction term in our models with the *pers_rank* index implied that quota significance increases as the system becomes more candidate oriented, tests of significance of difference showed insignificant differences. In all of our tests that compared between coefficients for the quota variable in candidate and programmatically oriented countries, there was no significant difference. In a test of difference of means for the mean coefficient size between the two groups, there was also no significant difference. That said, this test depended on an extremely small number of degrees of freedom (t(7)). We therefore have some reservations in using such a test to disregard any alternative findings.

## Limitations and future research

The goal of this study was to theorize and test whether an institutional factor conditions the effect of electoral gender quotas on women’s representation in parliament. Electoral systems vary considerably between programmatic and candidate orientations.

We found that electoral gender quotas interacted with electoral systems and that based on descriptive data and model output, we could infer different degrees of impact for different systems. In both systems, gender quotas correlated with an increased number of female parliamentarians. That said, the difference was more significant in candidate-oriented systems and was at a higher level of significance. Further tests of significance though for differences between coefficients imply that these conclusions should not be considered unequivocal.

Why would it be possible though that candidate-oriented systems might be more affected by quotas? We proposed several reasons above. First, women are already at a distinct disadvantage in these systems, so any policy to advance women will have a more significant impact than in other systems. Second, women’s difficulty in gaining elected positions in candidate-oriented systems was largely because a major hurdle to being elected is the nomination process, which legislative quotas override [21]. Third, because of the emphasis placed on individual candidates in candidate-oriented systems, gender quotas and increased female representation may have a more widespread impact in increasing the number of women representatives beyond those elected through quotas. Female role models in politics and in other fields can inspire other women to follow a given path [39, 42]. Finally, candidate-oriented systems are generally more competitive than programmatically oriented systems [41]. Perhaps quotas mitigate the competitiveness for women while leaving it at the same level for men.

These potential explanations for quota efficacy in candidate-orientated systems represent important points for future investigations and extensions of this study. Which one of the explanations we have given above are the most relevant? Another possible extension of this study would be to compare between these explanations and assess their relative strength. While these questions are beyond the scope of this study, their answers would help greatly to further clarify how different aspects of candidate-oriented systems can be harnessed to help promote gender equality.

This study also demonstrated a need for empirical exploration of the criticisms that have been made of quotas. The theoretical criticisms of quotas were cited as potential reasons why quotas might hinder the equalizing effect in candidate-oriented systems. In practice, there seems to be little cause for concern. In fact, candidate-oriented quotas seem to be particularly amenable to quota goals. Quotas correlate with a larger change in female political representation in systems that emphasize the individual candidate identity. Therefore, understanding under what conditions these criticisms are relevant in light of these findings could be of particular relevance to both advocates and challengers of gender quotas.

Finally, because quotas have a differential effect, depending on systems, we should perhaps explore the need to tailor quotas. In which candidate- and programmatically-oriented systems are gender quotas most effective and why? How can quotas be tailored to the different features of each system? Should different systems favor different quota mechanisms? Past research has focused on general good practices in quota policy, and on the contingent effect of quotas. Future studies should take on these directions for further research and best practice recommendations.

## Supporting information

S1 Table Country list by category. (DOCX)

S2 Table Descriptive statistics for countries by system orientation. (DOCX)
