# Supplementary material for: Personalism or party platform? Gender quotas and women’s representation under different electoral system orientations
Source: PLoS One. 2021 Sep 23;16(9):e0257665. doi: 10.1371/journal.pone.0257665 (PMC8459978; doi:10.1371/journal.pone.0257665)
Supplement: S1 Table — (DOCX) [file pone.0257665.s001.docx]

**Supporting Information**

**Table 1- Country List by category**

| **Programmatic Orientation, With Quotas** | **Programmatic Orientation, Without Quotas** | **Candidate-Oriented, With Quotas** | **Candidate-Oriented, Without Quotas** |
| --- | --- | --- | --- |
| Argentina  Austria  Belgium  Costa Rica  Croatia  Dominican Republic  El Salvador  Greece  Guatemala  Guyana  Honduras  Indonesia  Mexico  Moldova, Republic of  Norway  Panama  Paraguay  Peru  Poland  Portugal  Romania  Slovenia  South Africa  Sweden  Uruguay | Bulgaria  Cape Verde  Chile  Cyprus  Czech Republic  Denmark  Estonia  Finland  Latvia  Netherlands  Slovak Republic  Switzerland | Albania  Armenia  Australia  Bolivia  Canada  Colombia  France  Germany  Korea, Republic of  Lesotho  Malawi  Mongolia  United Kingdom | Botswana  Comoros  Georgia  Ghana  Hungary  India  Italy  Jamaica  Japan  Kenya  Lithuania  Luxembourg  Madagascar  Malaysia  Mauritius  New Zealand  Niger  Nigeria  Papua New Guinea  Philippines  Russian Federation  Ukraine  United States of America  Venezuela  Zambia  Zimbabwe |
