# Supplementary material for: Personalism or party platform? Gender quotas and women’s representation under different electoral system orientations
Source: PLoS One. 2021 Sep 23;16(9):e0257665. doi: 10.1371/journal.pone.0257665 (PMC8459978; doi:10.1371/journal.pone.0257665)
Supplement: S2 Table — (DOCX) [file pone.0257665.s002.docx]

**Supporting Information**

**Table 2- Descriptive statistics for the candidate and programmatically oriented categories**

|  | **Candidate-Oriented** | **Programmatically Oriented** |
| --- | --- | --- |
| Average orientation score | 9.9 | 2.54 |
| Standard deviation of orientation score | 0.69 | 1.50 |
| Countries without quotas | 26 | 11 |
| Countries with quotas | 13 | 26 |
| Average rate of female parliamentarians (%), without quotas | 15.7 | 25.20 |
| Average rate of female parliamentarians (%), with quotas | 24.7 | 27.20 |
